# Supplementary material for: CLOOME: contrastive learning unlocks bioimaging databases for queries with chemical structures
Source: Nat Commun. 2023 Nov 13;14:7339. doi: 10.1038/s41467-023-42328-w (PMC10643690; doi:10.1038/s41467-023-42328-w)
Supplement: Supplementary file 1 — Supplementary Information [file 41467_2023_42328_MOESM1_ESM.pdf]

Supplementary Information
CLOOME: contrastive learning unlocks bioimaging databases for queries with chemical structures

1 Supplementary Notes

1.1 Notation overview

Definition	Symbol/Notation	Dimension
molecule-perturbed microscopy image	x	image dimension, e.g., $320 \times 320 \times 5$
chemical structure of molecule	z	symbolic, e.g. graph
image embedding	$\mathbf{x}$	d
structure embedding	$\mathbf{z}$	d
stacked image embeddings	$\mathbf{X}$	$d \times N$
stacked structure embeddings	$\mathbf{Z}$	$d \times N$
stored image embeddings	$\mathbf{U}$	$d \times N$
stored structure embeddings	$\mathbf{V}$	$d \times N$
image-retrieved image embedding	$\mathbf{U}_{\mathbf{x}_i}$	d
structure-retrieved image embedding	$\mathbf{U}_{\mathbf{z}_i}$	d
image-retrieved structure embedding	$\mathbf{V}_{\mathbf{x}_i}$	d
structure-retrieved structure embedding	$\mathbf{V}_{\mathbf{z}_i}$	d
microscopy image encoder	$\boldsymbol{h}^x(.)$	$\mathbb{R}^{320 \times 320 \times 5} \rightarrow d$
molecule structure encoder	$\boldsymbol{h}^z(.)$	$\mathcal{M} \rightarrow d$
temperature parameter of the loss functions	τ	
scaling parameter of Hopfield net	β	
embedding dimension	d	
batch or dataset size	N	
chemical space	$\mathcal{M}$	
indices	i, j, n	

Supplementary Table 1: Symbols and notations used in this paper.

1.2 CLOOME hyperparameter search space

Below, we state the hyperparameter selection for the results reported in this study, based on performance in a validation set for each one of the downstream tasks. For the retrieval task, the model was trained for 70 epochs for the random split and for 60 epochs for the scaffold split, based on the top-1 accuracy in validation. For the zero-shot molecule classification, the selected models were trained for 63 and 57 epochs for the random and scaffold split, respectively. For the zero-shot mechanism of action classification, the selected models were trained for 70 and 69 epochs for the random and scaffold split, respectively.

1.2.1 Retrieval task hyperparameter search space

	Hyperparameter	Explored space
Learning	Optimizer	{AdamW}
	Learning rate	{0.0005, ***0.001***, 0.005}
	Scheduler	{Cosine annealing with restarts}
	Weight decay	{0.1}
	Batch size	{***256***, 512}
	Warm-up iterations	{10000, ***20000***}
	Initial inverse temperature	{**14.3**, 30}
	Learnable inverse temperature	{***True***, False}
Image encoder	Image resolution	{320, ***520***}
	Model	{ResNet50}
Structure encoder	Input size	{1024, ***8192***}
	Number of layers	{4}
	Layer dimension	{1024}
	Activation	{ReLU}
	Batch normalization	{False, ***True***}
Hopfield layers	β	{**-**, 8, 14.3, 22}
Embedding space	Number of dimensions	{512}

Supplementary Table 2: Considered hyperparameter space of CLOOME models. The selected configurations on manual search on validation set shown in bold for the random split and in italics for the scaffold split.

1.2.2 Bioactivity prediction hyperparameter search space

	Hyperparameter	Explored space
Learning	Optimizer	{AdamW}
	Learning rate	{0.0005, **0.001**, 0.005}
	Scheduler	{Cosine annealing with restarts}
	Weight decay	{0.1}
	Batch size	{**256**, 512}
	Warm-up iterations	{10000, **20000**}
	Initial inverse temperature	{14.3, **30**}
	Learnable inverse temperature	{True, **False**}
Image encoder	Image resolution	{**320**, 520}
	Model	{ResNet50}
Structure encoder	Input size	{1024}
	Number of layers	{4}
	Layer dimension	{1024}
	Activation	{ReLU}
	Batch normalization	{False, **True**}
Hopfield layers	β	{-, 8, 14.3, **22**}
Embedding space	Number of dimensions	{512}

Supplementary Table 3: Considered hyperparameter space of CLOOME models. The selected configurations based on manual search on validation set shown in bold.

1.2.3 Zero-shot molecule prediction hyperparameter search space

	Hyperparameter	Explored space
Learning	Optimizer	{AdamW}
	Learning rate	{0.0005, ***0.001***, 0.005}
	Scheduler	{Cosine annealing with restarts}
	Weight decay	{0.1}
	Batch size	{***256***, 512}
	Warm-up iterations	{10000, ***20000***}
	Inverse temperature	{***14.3***, 30}
Image encoder	Image resolution	{320, ***520***}
	Model	{ResNet50}
Structure encoder	Input size	{***1024***, 8192}
	Number of layers	{4}
	Layer dimension	{1024}
	Activation	{ReLU}
	Batch normalization	{False, ***True***}
Hopfield layers	β	{-, 8, 14.3, 22}
Embedding space	Number of dimensions	{512}

Supplementary Table 4: Considered hyperparameter space of CLOOME models. The selected configurations based on manual search on validation set shown in bold for the random split and in italics for the scaffold split.

1.2.4 Zero-shot MoA prediction hyperparameter search space

	Hyperparameter	Explored space
Learning	Optimizer	{AdamW}
	Learning rate	{0.0005, ***0.001***, 0.005}
	Scheduler	{Cosine annealing with restarts}
	Weight decay	{0.1}
	Batch size	{***256***, 512}
	Warm-up iterations	{10000, ***20000***}
	Inverse temperature	{***14.3***, 30}
Image encoder	Image resolution	{320, ***520***}
	Model	{ResNet50}
Structure encoder	Input size	{***1024***, 8192}
	Number of layers	{4}
	Layer dimension	{1024}
	Activation	{ReLU}
	Batch normalization	{False, ***True***}
Hopfield layers	β	{-, 8, 14.3, 22}
Embedding space	Number of dimensions	{512}

Supplementary Table 5: Considered hyperparameter space of CLOOME models. The selected configurations based on manual search on validation set shown in bold for the random split and in italics for the scaffold split.

1.3 Bilinear model hyperparameter search space

The hyperparameters of the bilinear models that yielded the results shown in this paper were also selected based on performance in a validation set, and are shown in Table 1.3. The selected models were trained for 52 and 54 epochs for the random and scaffold split, respectively.

	Hyperparameter	Explored space
Learning	Optimizer	{SGD, ***AdamW***}
	Learning rate	{**0.0005**, *0.001*}
	Scheduler	{No scheduler, ***Cosine annealing with restarts***}
	Weight decay	{0.1}
	Batch size	{**256**, *512*, 1024}
	Warm-up iterations	{5000, 10000, ***20000***}
Weight matrix	Size	{(1081, 8192)}

Supplementary Table 6: Considered hyperparameter space for the bilinear model. The selected configurations based on manual search on validation set shown in bold for the random split and in italics for the scaffold split.

1.4 Retrieval task results for sampled images or molecules

In Table 7, we report the Top-1, Top-5 and Top-10 accuracies in the structure and image retrieval, respectively, for a sampling rate of 1%, or equivalently, 1 matched example along with 99 un-matched ones – a setting often used to evaluate retrieval systems.

Split	Method	Top-k accuracy (%)					
		Top-1	95%-CI	Top-5	95%-CI	Top-10	95%-CI
Random	CLOOME (structure retr.)	**11.3**	[9.94, 12.7]	**21.7**	[19.9, 23.5]	**31.7**	[29.7, 33.7]
	CLOOME (image retr.)	**10.7**	[9.45, 12.1]	**22.0**	[20.3, 23.9]	**31.0**	[29.0, 33.0]
	Bilinear model (structure retr.)	5.48	[4.55, 6.54]	15.4	[13.9, 17.0]	22.1	[20.4, 24.0]
	Bilinear model (image retr.)	6.48	[5.47, 7.61]	16.0	[14.5, 17.7]	22.6	[20.9, 24.5]
	Random	0.992	[0.616, 1.51]	5.01	[4.12, 6.03]	10.0	[8.78, 11.4]
Scaffold	CLOOME (structure retr.)	**8.08**	[6.71, 9.64]	**18.6**	[16.6, 20.7]	**26.8**	[24.5, 29.2]
	CLOOME (image retr.)	**8.37**	[7.00, 9.95]	**17.7**	[15.8, 19.8]	**25.9**	[23.6, 28.3]
	Bilinear model (structure retr.)	4.36	[3.36 5.57]	11.9	[10.3, 13.8]	18.3	[16.3, 20.4]
	Bilinear model (image retr.)	4.58	[3.54, 5.81]	13.2	[11.4, 15.0]	19.8	[17.8, 22.0]
	Random	1.00	[0.549, 0.167]	5.01	[3.92, 6.28]	10.0	[8.49, 11.7]

Supplementary Table 7: Results for the retrieval task among 100 candidates. Given a molecule-perturbed microscopy image, the matched molecule must be selected from a set of candidates, and vice versa. Top-1, top-5 and top-10 accuracy in percentage are shown for a hold-out test set, along with the upper and lower limits for a 95% confidence interval (CI) ($n = 2,115$ for the random split and $n = 1,398$ for the scaffold split) on the resulting proportion. The best method in each category is marked in bold.

1.5 Downstream tasks evaluation with corrupted images

In this section, we evaluated the performance of CLOOME when carrying out different corruptions (see Figure 1), which were not considered during pre-training, to the test images. The goal of this evaluation is to assess its robustness to changes in the data distribution and simulate a scenario where the images used for inference might exhibit a domain shift to the images used during pre-training.

Different corruptions and their effects on performance metrics. In this experiment, we investigated the impact of the following transformations on performance metrics (see Table 8): random horizontal and vertical flipping, small rotation (from -10 to 10 degrees with respect to the center of the image), random horizontal and vertical flipping with small rotation, and large rotation (from -180 to 180 degrees). The performance metrics drop only slightly for most tasks. In fact, for the cross-modal retrieval tasks (Tables 8 and 9), the performance on corrupted image remains mostly within the confidence intervals of the previous evaluation with the original images. As shown in Figure 1, rotations introduce wedge-like structures that are added to fill the rectangular shape, which could explain the lower accuracy. As expected, introducing different image transformations not considered during training slightly affects the performance metrics.

Further experiments with corrupted images. We selected one of these transformations (specifically the random horizontal and vertical flipping together with small rotation), to show the effect of using corrupted images in the rest of the downstream tasks. Regarding *bioactivity prediction* performance, shown in Table 10, the mean AUC changes only from 0.714 to 0.713. As shown in Tables 11 and 12, or the *zero-shot tasks*, the considered distortions affect the performance more than in the retrieval and linear probing tasks. A possible

explanation is that image embeddings corresponding to cells treated with different molecules are closer to each other than their corresponding structure embeddings. If this is the case, corrupting the images will have a larger impact in image-to-image (i.e. zero-shot) tasks than in cross-modal tasks (i.e. retrieval).

Image transformation	Method	Accuracy[%]					
		Top-1	95% CI	Top-5	95% CI	Top-10	95% CI
Random flip	CLOOME (structure retr.)	3.22	[2.51, 4.06]	6.86	[5.82, 8.02]	8.61	[7.44, 9.88]
	CLOOME (image retr.)	2.55	[1.92, 3.32]	6.48	[5.47, 7.61]	8.79	[7.62, 10.1]
Random flip + small rotation	CLOOME (structure retr.)	2.98	[2.30, 3.80]	6.76	[5.73, 7.92]	8.84	[7.67, 10.1]
	CLOOME (image retr.)	2.60	[1.96, 3.37]	6.62	[5.60, 7.76]	8.61	[7.44, 9.88]
Small rotation	CLOOME (structure retr.)	3.59	[2.84, 4.48]	7.85	[6.74, 9.08]	9.46	[8.24, 10.8]
	CLOOME (image retr.)	2.98	[2.30, 3.80]	8.18	[7.05, 9.43]	9.60	[8.38, 10.9]
Large rotation	CLOOME (structure retr.)	3.07	[2.38, 3.90]	6.57	[5.55, 7.71]	8.37	[7.22, 9.63]
	CLOOME (image retr.)	2.65	[2.01, 3.42]	6.67	[5.64, 7.81]	8.51	[7.36, 9.78]

Supplementary Table 8: Results of CLOOME for the retrieval task when performing different types of transformations to the test images for a random split. Top-1, top-5 and top-10 accuracy in percentage are shown for a hold-out test set, along with the upper and lower limits for a 95% confidence interval (CI) (n = 2,115 samples) on the resulting proportion.

Split	Method	Accuracy[%]					
		Top-1	95% CI	Top-5	95% CI	Top-10	95% CI
Scaffold	CLOOME (structure retr.)	2.15	[1.45, 3.05]	5.44	[4.31, 6.76]	6.94	[5.66, 8.40]
	CLOOME (image retr.)	2.58	[1.81, 3.55]	6.15	[4.95, 7.54]	7.44	[6.12, 8.94]

Supplementary Table 9: Results of CLOOME for the retrieval task with corrupted images (by random flipping and rotation from -10 to 10 degrees) using a scaffold split. Top-1, top-5 and top-10 accuracy in percentage are shown for a hold-out test set, along with the upper and lower limits for a 95% confidence interval (CI) (n = 1,398 samples) on the resulting proportion.

Type	Method	AUC	F1	AUC >0.9	AUC >0.8	AUC >0.7
Linear probing on self-supervised	CLOOME	0.713±0.20	0.397±0.33	59	83	108

Supplementary Table 10: Linear probing evaluation results of the learned representations with corrupted images (by random flipping and rotation from -10 to 10 degrees).the performance metrics area under the receiver operating characteristic curve (AUC) and F1-score are shown, along with their standard deviation (n = 209 tasks), and the number of tasks with an AUC higher than 0.9, 0.8 and 0.7

Split	Method	Accuracy[%]					
		Top-1	95% CI	Top-5	95% CI	Top-10	95% CI
Random	CLOOME (structure retr.)	13.5	[13.2, 13.8]	31.8	[31.4, 32.2]	44.1	[43.6, 44.6]
Scaffold	CLOOME (image retr.)	16.7	[16.2, 17.1]	39.4	[38.8, 40.0]	53.5	[53.0, 54.1]

Supplementary Table 11: Results of CLOOME for the zero-shot image-to-image molecule classification task with corrupted images (by random flipping and rotation from -10 to 10 degrees). Top-1, top-5 and top-10 accuracy in percentage are shown for a hold-out test set, along with the upper and lower limits for a 95% confidence interval (CI) (n = 43, 778 for the random split and n = 28, 248 for the scaffold split) on the resulting proportion.

Split	Method	Accuracy[%]					
		Top-1	95% CI	Top-5	95% CI	Top-10	95% CI
Random	CLOOME	9.59	[8.98, 10.2]	28.2	[27.2, 29.1]	40.4	[39.3, 41.4]
Scaffold	CLOOME	14.6	[13.5, 15.7]	40.2	[38.7, 41.7]	52.8	[51.3, 54.4]

Supplementary Table 12: Results of CLOOME for the zero-shot image-to-image MoA classification task with corrupted images (by random flipping and rotation from -10 to 10 degrees). Top-1, top-5 and top-10 accuracy in percentage are shown for a hold-out test set, along with the upper and lower limits for a 95% confidence interval (CI) (n = 8, 826 for the random split and n = 4, 056 for the scaffold split) on the resulting proportion.

2 Supplementary Figures

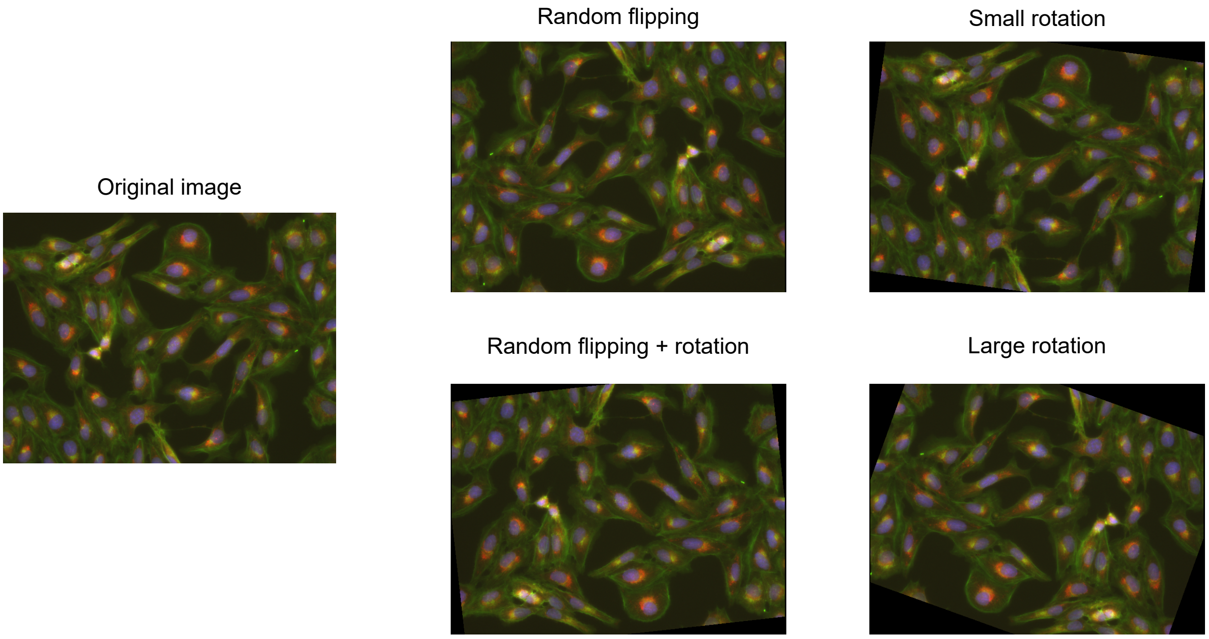

Supplementary Figure 1: Visualization of the different transformations applied to the images. As visible in the two images on the right hand side, rotations introduce artifacts, concretely wedge-like structures at the image borders, which can have an effect on performance metrics.
